# Effectiveness of combined exercise training for hypertension management: a systematic review and meta-analysis of randomized controlled trials

**DOI:** 10.11604/pamj.2026.53.111.48696

**Published:** 2026-03-04

**Authors:** Chaimaa Chourite, Hanane Belmouss, Mohammed Fedouache, Ismail Challal, Mohammed Zaguiri, Mustapha Mouilly

**Affiliations:** 1 Interdisciplinary Laboratory of Sport Sciences, Institute of Sport Professions, Ibn Tofail University, Kenitra, Morocco,; 2 National School of Applied Sciences, Cadi Ayyad University, Marrakech, Morocco,; 3 Department of Biology, Ibn Tofail University, Kenitra, Morocco,; 4 Higher Institute of Nursing and Health Techniques Professions, Casablanca, Morocco

**Keywords:** Aerobic training, combined exercise, hypertension, meta-analysis, randomized controlled trials, resistance training

## Abstract

**Introduction:**

hypertension is a leading global health issue with poor control despite pharmacological treatments. Combined exercise is a promising non-pharmacological strategy. This systematic review aimed to evaluate the effectiveness of combined aerobic and resistance training in reducing blood pressure (BP) among adults with hypertension.

**Methods:**

a systematic literature review and meta-analysis were conducted in PubMed, Scopus, Web of Science, Science Direct and Google Scholar to identify randomized controlled trials (RCTs) involving hypertensive adults undergoing combined exercise (CE) interventions. Study selection, data extraction, and risk of bias assessment were performed following Preferred Reporting Items for Systematic Reviews and Meta- Analyses (PRISMA) guidelines.

**Results:**

we included five RCTs in this study, four RCTs with a pooled population of 154 participants showed a significant reduction in systolic BP (SBP) favoring combined exercise (MD = -8.26 mmHg; 95% CI [-13.92, -2.61]; I^2^= 54.8%). Five studies including 182 participants demonstrated a significant decrease in diastolic BP (DBP) (MD = -5.87 mmHg; 95% CI [-7.91, -3.83]; I^2^= 0%). However, for mean arterial pressure (MAP), based on three RCTs the meta-analysis revealed no significant difference between the CE and control groups (MD=-1.03mmHg;95% CI [-6.36,4.30]; I^2^=15%).

**Conclusion:**

our findings suggest that combined exercise may serve as an effective complementary intervention to reduce both SBP and DBP in adults with hypertension.

## Introduction

Hypertension remains one of the most prevalent chronic diseases worldwide and continues to pose a major challenge to public health systems [1]. It affected more than 1 billion adults globally in 2016, a number projected to exceed 1.5 billion by 2025 [2]. It is characterized by persistently elevated arterial pressure that exerts continuous mechanical stress on the cardiovascular system, leading to vascular remodeling, endothelial dysfunction [3,4]. As a result, hypertension is a well-recognized risk factor for stroke, coronary artery disease, heart failure, chronic kidney disease, and premature mortality [5,6]. Studies suggest that the etiology of hypertension is multifactorial, involving genetic, environmental, and behavioral determinants [7,8]. Aging, obesity, high dietary sodium intake, physical inactivity, and chronic psychosocial stress interact to elevate vascular resistance and impair baroreflex sensitivity [9]. Age-related arterial stiffening and oxidative stress further amplify this burden, making older adults particularly susceptible to persistent hypertension [10]. Beyond its physiological consequences, hypertension imposes a heavy socioeconomic cost through increased healthcare expenditure and loss of productivity, reinforcing the necessity of sustainable preventive strategies [11]. Despite substantial advances in pharmacotherapy, fewer than 20% of diagnosed individuals achieve optimal blood pressure (BP) control [12]. A recent study found that physical activity was the least frequently prescribed lifestyle intervention, despite being a core recommendation for BP control [13]. Additionally, a community-based study conducted in Akure South, Nigeria, reported a hypertension prevalence of 27.9%, with nearly half (47.2%) of the population exhibiting physical inactivity.

Significant predictors of hypertension included age, obesity, diabetes, dyslipidemia, and low physical activity, highlighting the urgent need for early detection and preventive community-based strategies [14]. Non-pharmacological interventions have thus emerged as indispensable components of comprehensive hypertension management. Among them, physical exercise represents one of the most effective, accessible, and low-cost strategies for BP control [15-17]. Regular physical activity promotes favorable hemodynamic adaptations, including improved endothelial function, enhanced nitric oxide bioavailability, reduced sympathetic activity, and greater arterial compliance [18]. Aerobic exercise such as walking, cycling, or swimming typically lowers systolic blood pressure (SBP) by 6 to 10 mmHg and diastolic blood pressure (DBP) by 3 to 6 mmHg through mechanisms of vasodilation and improved cardiac efficiency [19,20]. Resistance training, characterized by muscle contractions against external loads, complements these effects by increasing muscular strength and lean mass while improving metabolic and autonomic profiles [21]. When aerobic and resistance modalities are integrated within the same training regimen known as combined or concurrent exercise (CE) they can elicit additive or synergistic cardiovascular adaptations. Aerobic components predominantly enhance endothelial and autonomic regulation, whereas resistance components strengthen peripheral musculature, improve glucose and lipid metabolism, and mitigate vascular stiffness [22,23]. Consequently, CE holds particular promise for adults and older individuals who often present both elevated vascular resistance and declining muscle mass [24]. Given the growing prevalence of hypertension and the need for evidence-based interventions, the present systematic review and meta-analysis aimed to evaluate the effectiveness of combined aerobic and resistance exercise training in reducing BP among adults with hypertension.

## Methods

**Figure 1:**
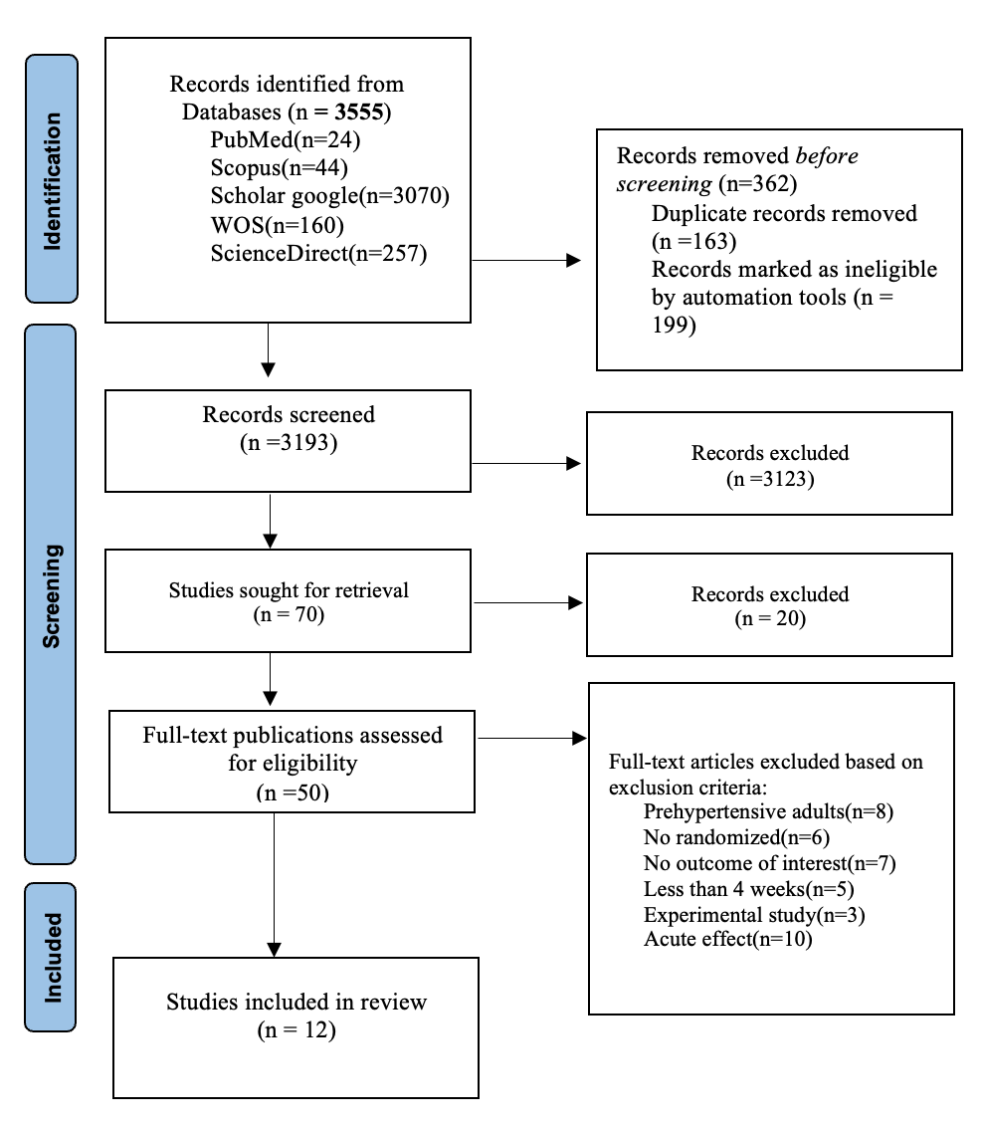
PRISMA flow diagram illustrating the identification, screening, eligibility assessment, and inclusion process of RCTs in this systematic review on combined exercise and hypertension management

**Literature research:** this systematic review of randomized controlled trials followed the Preferred Reporting Items for Systematic Review and Meta-Analyses checklist 2020 (PRISMA) (Figure 1) [25]. The primary objective of our research was to identify scientific evidence of the efficacy of combined physical exercise in managing blood pressure among individuals diagnosed with hypertension. In this international systematic review of randomized controlled trials, we conducted electronic searches of PubMed, Scopus, Google Scholar, Web of Science, and Science Direct with no restriction applied. The search strategy used keywords, MeSH terms and Boolean connectors including Mesh terms: “Combined exercise” AND “Blood pressure” AND “Hypertension” AND “Randomized controlled trial”.

**Figure 2:**
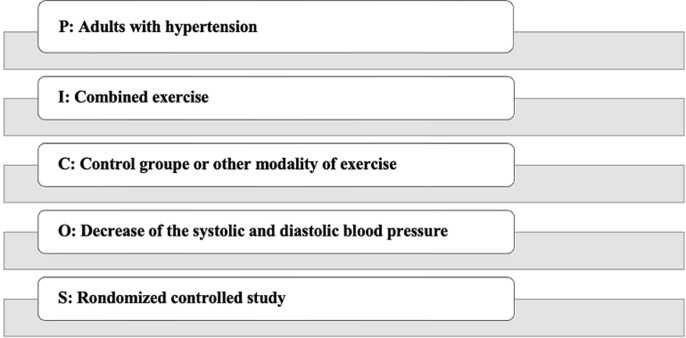
PICOS framework defining the eligibility criteria for inclusion in this systematic review and meta-analysis evaluating the effects of combined aerobic and resistance exercise versus control group

**Selection of studies for eligibility criteria:** studies were eligible for inclusion if they were randomized controlled trials (RCTs) that investigated the effects of combined aerobic and resistance training interventions in adults diagnosed with hypertension. Eligible trials were required to report blood pressure outcomes as a primary or secondary endpoint, classified according to the International Society of Hypertension guidelines. To ensure sufficient exposure for physiological adaptation, only interventions lasting at least four weeks were included. Studies were excluded if they focused on prehypertensive populations, patients with resistant or pulmonary hypertension, pregnant women, or pediatric participants. Trials conducted in animal models and articles not published in English were also excluded from this review. Data extraction followed the PICOS strategy (Figure 2) [26], encompassing various characteristics such as author details, publication year, study location (Table 1, Table 1.1). Additionally, information on sample characteristics (size, age, gender) (Table 2) intervention specifics (type, duration, frequency) and key outcomes (effects on SBP, DBP and MAP) (Table 3). Of the 12 RCTs meeting our eligibility criteria, all were synthesized qualitatively, whereas a prespecified subset (n= 5) was entered into the meta-analysis. Trials were pooled only when they investigated combined aerobic resistance exercise versus a control, reported systolic, diastolic and mean blood pressure at commensurate post-intervention time points using comparable definitions, and provided sufficient statistics for aggregation (group means, standard deviations, and sample sizes).

**Table 1:** general characteristics of randomized controlled trials included in this systematic review evaluating the effect of combined aerobic and resistance exercise on blood pressure in adults with stage 1-2 hypertension (N = 547)

Authors	Study design	Sample size	Gender	Age in years	Stage of hypertension	Measurement method
Herawati *et al*. 2025 Indonesia	RCT with 4 parallel groups	87 CG:24 BE:21 HIBIT:20 CE;22	84F/3M	59,6± 9,2	Stage 1 and 2	Automated digital BP monitor OMRON SEM-1
Alemayehu 2023 Ethiopia	RCT with 4 parallel groups	46 CG: 12 AG: 11 RG: 11 CE: 12	46M	45,28± 7,44	Stage 1 and 2	Automated Sphygmacor XCEL AtCor Medical, Itasca, IL, USA
Sardeli *et al*. 2022 Iran	RCT with 2 parallel groups	40 CG:20 CE:20	40F	50-70	Stage 1 and 2	Automated BP device OMRON HEM-7130
Da silva *et al*. 2022 Brazil	RCT with 3 parallel groups	60 CG: 20 CE: 20 MP: 20	60F	30 to 59	Stage 1	Ambulatory blood pressure monitor, modèle Dyna-Mapa
Ruangthai *et al*. 2020 Thailand	RCT with 3 parallel groups	41 CG: 12 Let: 16 Wet:13	20F/11M	66,4± 5,8	Stage 1	Automated oscillometric device OMRON model HEM 705-CP, Tokyo, Japan
Oliveira *et al*. 2019 Brazil	RCT with 4 parallel groups	60 CG:15 RG:15 AG:15 CE:15	36F/24M	30-59	Stage 1	Automated oscillometric device

Abbreviations: AG: aerobic group; BE: breathing exercise; F: Female; CE: combined exercise; CG: control group; DBP: diastolic blood pressure; RCT: randomized controlled trials; HIBIT: high-intensity bodyweight interval training; M: male; MAP: mean arterial pressure; NA: not available; RG: resistance group; SBP: systolic blood pressure

**Table 1.1:** general characteristics of randomized controlled trials included in this systematic review evaluating the effect of combined aerobic and resistance exercise on blood pressure in adults with stage 1-2 hypertension (N = 547)

Authors	Study design	Sample size	Gender	Age in years	Stage of hypertension	Measurement method
Ruangthai *et al*. 2019 Thailand	RCT with 4 parallel groups	54 CG: 12 ET: 13 ST: 13 CE: 16	32F/6M	67.4± 5.8	Stage 1	Oscillometric semiautomatic device Omron model HEM 705-CP, Omron Corporation, Tokyo, Japan
Leandro *et al*. 2019 Brazil	RCT with 3 parallel groups	24 AST:8 SAT:8 ASAT:8	24F	64± 0.19	Stage 1 and 2	Auscultatory method
Schroeder *et al*. 2019 USA	RCT with 4 parallel groups	69 CG: 17 AG: 17 RG: 17 CE: 18	42F/27M	58± 7	Stage 1	Sphygmocor XCEL, AtCor Medical, Itasca, IL, USA
Masroor *et al*. 2018 India	RCT with 2 parallel groups	28 CG: 13 CE: 15	28F	40.54±4.2	Stage 1 and 2	Manual sphygmomanometer
Lima *et al*. 2017 Brazil	RCT with 3 parallel groups	44 CG: 20 AT: 20 CE: 20	7F/37M	67.8± 4.3	Stage1	ABP Meditech KFT Ulloi, H-1191, Budapest, Hongrie
Son *et al*. 2016 South Korea	RCT with 2 parallel groups	20 CT: 10 CE: 10	20F	74.7± 2	Stage 1	Standard BP measurements

Abbreviations: AG: aerobic group; BE: breathing exercise; CE: combined exercise; CG: control group; DBP: diastolic blood pressure; HIBIT: high-intensity bodyweight interval training; MAP: mean arterial pressure; RG: resistance group; SBP: systolic blood pressure; RCT: randomized controlled trials

**Table 2:** characteristics of combined aerobic and resistance exercise interventions (type, duration, frequency, and session length) in randomized controlled trials assessing blood pressure outcomes in hypertensive adults (N= 12 RCTs)

Study	Type of intervention	Duration intervention	Frequency intervention	Time/session
Herawati *et al*. 2025	Combined exercise HIBIT and breathing exercise	10 Weeks	3 sessions per week	60 min
Alemayehu *et al*. 2023	Combined exercise vs aerobic group and resistance group	12 Weeks	3 sessions per week	60 min
Sardeli *et al*. 2022	Combined training: aerobic and resistance	8 weeks	3 sessions per week	60 min
Da Silva *et al*. 2022	Mat pilates group with aerobic exercise vs Mat-pilates group and control group	16 Weeks	2 sessions per week	60 min
Ruangthai *et al*. 2020	Combined water and land-based exercise vs aerobic exercise	12 weeks	3 sessions per week	60 min
Olivera *et al*. 2019	Combined exercise vs aerobic, resistance, and control	12 weeks	3 sessions per week	40-60 min
Ruangthai *et al*. 2019	Combined exercise vs aerobic training and resistance training	12 weeks	3 sessions per week	60 min
Leandro *et al*. 2019	Combined exercise vs aerobic and resistance and control groups	8 weeks	3 sessions per week	60 min
Schroeder *et al*. 2019	Combined exercise vs control group	4 weeks	3 sessions per week	60 min
Masroor *et al*. 2018	Combined exercise vs aerobic training and control group	10 weeks	3 sessions per week	60 min
Lima *et al*. 2017	Combined aerobic and resistance band exercise	12 weeks	3 sessions per week	60 min
Son *et al*. 2016	Combined exercise vs aerobic training and control group	12 weeks	3 sessions per week	60 min

**Table 3:** changes in systolic blood pressure, diastolic blood pressure, and mean arterial pressure, before and after combined exercise interventions in randomized controlled trials involving adults with stage 1-2 hypertension (N= 547)

Studies	Difference in SBP		Difference in DBP		Difference in MAP	
	Before intervention	After intervention	Before intervention	After intervention	Before intervention	After intervention
Herawati *et al*. 2025	CG: 151 ± 11BE: 159 ± 11HIBIT: 160 ± 17 CE: 152 ± 2	CG: 151 ± 15 BE: 136 ± 11HIBIT: 141 ± 14CE: 137 ± 6^a^	CG: 86 ± 7BE: 93 ± 9HIBIT:96 ± 11CE: 88 ± 8	CG: 86 ± 7BE: 83 ± 6HIBIT: 82 ± 6CE: 80 ± 3^a^	NA	
Alemayehu and Teferi, 2023	CG:154±5.04 CE:154.17±5	CE Vs CG: -12.67 ± 2.21^a^	CG:92.58±6.23 CE:93.5±6.8	CE Vs CG: -9 ± 2.28^a^	NA	
Sardeli *et al*. 2022	CG:132 ±23CE:133 ±15	CG:132.3 ±14.4 CE:129.9 ±14.7	CG:79.5 ±9.5 CE:86 ±8.2	CG:84.4±10.2CE:80 ±10.3^a^	CG:97±12.8CE:101.7±8.7	CG:96.8±10.6CE:99.6±10.9^b^
Da Silva *et al*. 2022	CG: 123.3 ± 11.7CE: 124 ± 8.9	CG: 123 ± 12.6CE: 118.9 ± 9^b^	CG78.6 ± 8.9CE: 77.8 ± 6.3	CG:78.4 ± 8.9CE: 75.1 ± 6	CG:90.4±7.6CE:92±8.4	CG:93.3±9.5 CE:89.7±5.2 ^b^
Ruangthai *et al*. 2020	LET: -11.6 and -10.6a WET: -6.5 and -7.6^c^		LET: -8.1a WET: NA		NA	
Olivera *et al*. 2019	CG:143.5 RG:141.5 AG:143.1 CE:142.8	CG:142.2 RG:134.1 AG:134.9 CE:132.3^b^	CE: 90.5 RG:91.2 CG: 91 CE:89.8	CE:84.4 RG:86.2 CG: 90.2 CE:85.1^a^	CG: NA CE: -5mmHg ^b^	
Ruangthai and Phoemsapthawee, 2019	CG:140.6 ± 18.2 CE:142 ± 12.2	CG: 142.1 ± 13.1 CE: 130.4 ± 9.4^a^	CG:82.5± 10.1 CE: 81.7 ± 5.6	CG:82.1±12.6 CE:76.9 ± 7.4 ^c^	CG:93.4±31.4 CE:101.8±6.6	CG:96.8±32.4 CE:94.8±7.2^a^
Leandro *et al*. 2019	SAT: 113.8 ± 7.1 TSA:130.4± 23.47 ASAT:142.96±20.91	SAT:113.4± 4.77 TSA:133.4± 7.82 ASAT:124± 9.95 ^b^	SAT:57.4±14.35 TSA: 71 ± 7.69 ASAT:79.48±19.48	SAT:53.33±21.83 TSA:71 ± 4 ASAT:61.68 ± 11.07 ^c^	SAT:77.33±12.11 TSA:88.30 ± 11.73 ASAT:98.4± 15.42	SAT:66.53±12.46^c^ TSA:89.4 ± 8.76 ASAT:83.44± 7.92 ^c^
Schroeder *et al*. 2019	-1 within group changes ^c^		- 4 within group changes ^c^		NA	
Masroor *et al*., 2018	CG:149.4±11.57 CE: 141.6 ±4.63	CG:149±10.53 CE: 122.5 ±9.16 ^b^	CG:85.0±3.75 CE: 84.6± 6.54	CG:86.3±6.35 CE:77.4 ±5.74 b	NA	
Lima *et al*., 2017	CG: 127.3 ± 11 CE: 128.5 ± 11.7	CG: 133 CE: 12 ^b^	CG: 72.8 ± 8.1 CE: 75.7 ± 5.5	CG: 73.5 CE: 71.9^c^	NA	
Son *et al*. 2016	CG: 149 ± 2 CE: 152±2	CG: 150 ± 2 CE: 95± 3^c^	CG: 95± 3 CE: 95± 3	CG: 96 ± 2 CE: 84 ± 1.34 ^c^	CG:110±3 CE:112±4	CG:96.8±10.6 CE:99.6±10.9^a^

Abbreviations: AG: aerobic group; ASAT: aerobic strength aerobic training; AST: aerobic strength training; BE: breathing exercise; CE: combined exercise; CG: control group; HIBIT: high-intensity bodyweight interval training; NA: not available; RCT: randomized controlled trials; RG: resistance group; SAT: strength aerobic training; systolic blood pressure (SBP); diastolic blood pressure (DBP); mean arterial pressure (MAP) Statistical notes: a P<.001; b P<.005; c P<.05

**Study selection:** a total of 3190 studies were identified by electronic database searching and loaded to the Zotero reference manager. After removal of duplicates, a total of 12 studies with 547 patients (393 female and 154 male) are currently included for data extraction. These studies were published between 2015 and 2025 and were conducted in various countries: Indonesia (n=1) [27], Brazil (n=4) [23,28-30], Thailand (n=2) [31,32], Iran (n=1), Ethiopia (n=1) [33], the United States (n=1) [34], India (n=1) [35] and South Korea (n=1) [36] (Table 1, Table 1.1). During the study identification and selection phase, a total of 3555 articles were initially located across various databases. Following the elimination of 361 duplicates, 3194 studies underwent a review of their titles and abstracts to determine compliance with the inclusion criteria. Subsequently, a total of 12 randomized controlled trials met all eligibility criteria, leading to a comprehensive full-text evaluation.

**Appraisal of the risk of bias of included studies:** to evaluate the risk of bias in included studies, the tool recommended by the Cochrane Manual of Systematic Reviews of Interventions [37] was utilized. This tool assesses five domains, with each domain evaluated as “High risk”, “Low risk”, or “Uncertain risk” (Table 4). The domains considered for the risk of bias assessment include: bias arising from the randomization process, deviations from intended interventions, missing outcome data, bias in the measurement of the outcome, and bias in the selection of the reported result [38]. Two authors (CC and HB) independently conducted the literature search across the selected databases. Each author applied the predefined search strategy separately on PubMed, Scopus, Google Scholar, Web of Science and ScienceDirect to ensure comprehensive coverage and minimize selection bias. Any discrepancies in the retrieved records were discussed and resolved through consensus, thereby enhancing the methodological rigor and reliability of the review process.

**Table 4:** Cochrane risk of bias rob risk of bias assessment of included randomized controlled trials evaluating combined exercise effects on blood pressure in hypertensive adults, using the Cochrane risk of bias tool (N = 12 RCTs)

Study	Randomization	Deviations from intended interventions	Missing outcome data	Measurement of the outcome	Selection of the reported result	Overall risk of bias
Herawati *et al*. 2025	Low risk	Low risk	Low risk	Low risk	Low risk	Low
Alemayehu and Teferi *et al*. 2023	Low risk	Low risk	Low risk	Low risk	Low risk	Low
Sardeli *et al*. 2022	Low risk	Low risk	Low risk	Low risk	Low risk	Low
Da Silva Almeida *et al*. 2022	Low risk	Low risk	Low risk	Low risk	Low risk	Low
Ruangthai *et al*. 2020	Low risk	Low risk	Low risk	Low risk	Low risk	Low
Oliveira *et al*. 2019	Low risk	Low risk	Low risk	Low risk	Low risk	Low
Ruangthai *et al*. 2019	Low risk	Low risk	Low risk	Low risk	Low risk	Low
Leandro *et al*. 2019	Low risk	Low risk	Low risk	Low risk	Low risk	Low
Schroeder *et al*. 2019	Low risk	Uncertain	Uncertain	Low risk	Low risk	Moderate
Masroor *et al*. 2018	Low risk	Low risk	Low risk	Low risk	Low risk	Low
Lima *et al*. 2017	Low risk	Uncertain	Uncertain	Uncertain	Uncertain	Moderate
Son *et al*. 2016	Low risk	Low risk	Low risk	Low risk	Low risk	Low

Low risk: sufficient information, rigorous methodology, and clear results; Uncertain: some methodological uncertainties; High risk: significant methodological flaws.

**Figure 3:**
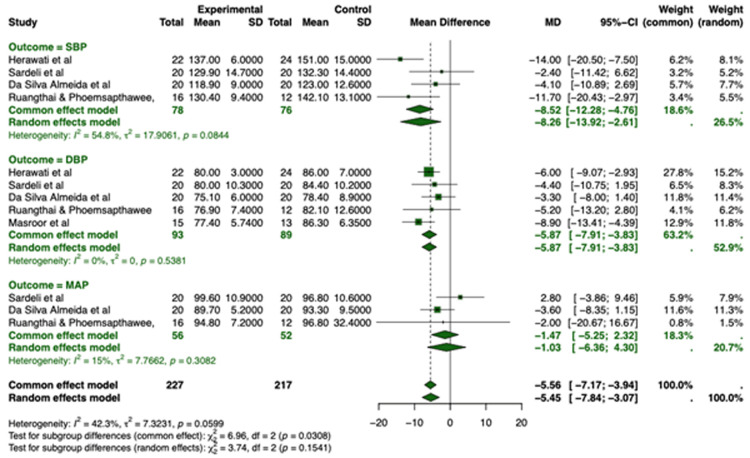
forest plots from random-effects meta-analysis showing the pooled effects of combined aerobic and resistance exercise versus control on (A) systolic blood pressure (4 RCTs; N = 154), (B) diastolic blood pressure (5 RCTs; N = 182), and (C) mean arterial pressure (3 RCTs; N = 108) in adults with hypertension

**Data synthesis and analysis:** the meta-analysis focused on the key outcomes of interest: SBP, DBP and MAP, utilizing post-intervention data in Figure 3. All meta-analyses were independently performed by two authors (CC and MM). The analyses were conducted in R (version 4.5.1) using the meta and metafor packages. A random-effects model was employed to account for expected between-study heterogeneity. Statistical heterogeneity was quantified using the I^2^statistic, with values ≥50% considered indicative of substantial heterogeneity. The significance level was set at P < 0.05 was applied, and forest plots were generated to visually represent the meta-analysis results.

## Results

**Characteristics of the included studies:** the main characteristics of the 12 RCTs included in this systematic review are presented in Table 1, Table 1.1. The total sample comprised 547 hypertensive adults with a mean age of 58 years [27,35], both men and women were represented, although several trials included predominantly female samples [32,36]. Blood pressure was assessed using validated methods, including automated oscillometric devices and ambulatory monitors [29,31]. All trials focused on individuals with stage 1 or stage 2 hypertension [23,28,33] providing a comprehensive foundation for evaluating the non-pharmacological impact of combined exercise on blood pressure regulation in adult hypertensive populations.

**Risk of bias:** the risk of bias across the included RCTs was generally low, indicating strong methodological rigor. Ten studies were rated as having low risk of bias across all five domains, including randomization, adherence to intended interventions, missing outcome data, outcome measurement, and selective reporting (Table 4). Conversely, two studies exhibited some concerns, particularly related to deviations from intended interventions, missing data, and uncertainty in outcome reporting, resulting in an overall moderate risk of bias [29,34]. Nevertheless, none of the included studies were judged to have a high risk of bias, which strengthens the overall reliability of the review´s findings.

**Figure 4:**
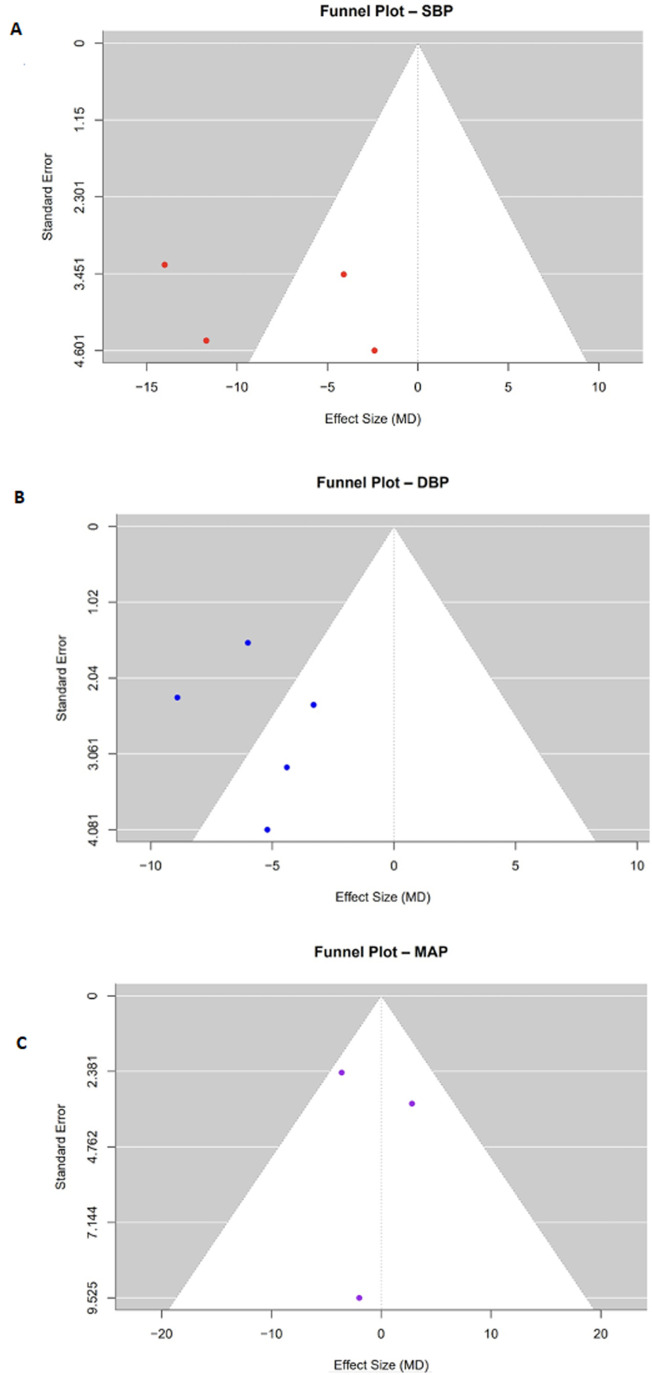
funnel plots assessing publication bias in RCTs included in the meta-analysis of combined exercise interventions on (A) systolic blood pressure, (B) diastolic blood pressure, and (C) mean arterial pressure in hypertensive adults

**Qualitative synthesis:** across twelve randomized controlled trials, combined exercise interventions consistently demonstrated significant improvements in BP among hypertensive adults. Combined exercise was associated with reductions in both SBP and DBP, with statistical significance reported in each case (P <.05 to P <.001). Notably, CE interventions achieved reductions in SBP ranging from approximately 9 to 30 mmHg [31,35] and DBP reductions from 3 to 14 mmHg, compared to baseline or control. For instance, two studies reported SBP declines of 15 mmHg and 12.7 mmHg respectively [27,33], while three studies demonstrated concurrent improvements in DBP, often exceeding 7mmHg [23,36]. These effects were observed across various populations, age groups, and durations, reinforcing the robustness of CE as a non-pharmacological strategy for BP management.

**Meta-analysis of systolic blood pressure:** four randomized controlled trials evaluated the effect of combined exercise training on SBP, covering 154 participants [27,28,31,39]. The pooled analysis demonstrated a significant reduction in SBP favoring the experimental group compared to controls (MD = -8.26 mmHg, 95% CI [-13.92, -2.61], I^2^= 54.8%, P=.084) (Figure 3). While the overall effect was robust, moderate heterogeneity was detected across studies, suggesting variability in intervention protocols or population characteristics. Sensitivity analyses confirmed the consistency of the effect, reinforcing the clinical relevance of SBP reduction with combined training. Sensitivity analysis conducted by excluding one moderate RCTs [35] while retraining four high-quality trials confirmed that there were no significant differences in SBP measurements between the two groups.

**Meta-analysis of diastolic blood pressure:** five RCTs (182 participants) reported DBP outcomes [27,28,31,35,39]. The pooled results revealed a significant reduction in DBP in the intervention groups compared to controls (MD = -5.87 mmHg, 95% CI [-7.91, -3.83], I^2^= 0%, P=.538) (Figure 3). Notably, the absence of heterogeneity strengthens the reliability of this finding, indicating a consistent benefit of combined exercise training in lowering DBP across studies.

**Meta-analysis of mean arterial pressure:** three RCTs [28,31,39] assessed MAP as an outcome and comprising 108 participants. The meta-analysis showed no significant difference between the combined exercise and control groups (MD = -1.03 mmHg, 95% CI [-6.36, 4.30], I^2^= 15%, P=.308) (Figure 3). Although the point estimate suggested a trend toward reduction, the wide confidence interval and the lack of statistical significance indicate insufficient evidence to conclude a clear effect of combined exercise on MAP.

**Publication bias assessment:** potential publication bias was assessed using visual inspection of funnel plots, which appeared symmetrical. This observation indicates that publication bias is unlikely to have materially affected the pooled estimates in this meta-analysis (Figure 4).

## Discussion

The present systematic review and meta-analysis aimed to evaluate the effectiveness of combined aerobic and resistance exercise training in reducing blood pressure among adults with hypertension. Twelve RCTs met eligibility criteria; five contributed to the meta-analysis, while seven were not pooled owing to missing data. By synthesizing data from the five RCTs conducted across diverse populations, this study suggested that combined exercise leads to clinically significant reductions in BP parameters. The most salient results showed an average decrease of -8.26 mmHg in SBP and -5.87 mmHg in DBP compared with control groups. The pooled analysis of four randomized controlled trials including 182 participants demonstrated a significant decline in SBP (MD = -8.26 mmHg). This magnitude of reduction is clinically meaningful and comparable to that obtained with first-line antihypertensive drugs [40]. Epidemiological evidence indicates that every 5 mmHg decrease in SBP corresponds to roughly a 10% lower risk of major cardiovascular events, emphasizing the public-health relevance of these findings [41]. The moderate heterogeneity observed likely reflects differences in training intensity, duration, and participant characteristics; however, the consistent direction of the effect and stability under sensitivity analysis confirm the robustness of the antihypertensive response to combined exercise. The reduction aligns with prior meta-analyses reporting mean SBP decreases of about 11 mmHg following similar interventions in hypertensive adults [42,43], supporting the reproducibility of this benefit across study designs and populations. Furthermore, studies in specific groups reinforce the general trend. Xi *et al*. reported a modest but statistically significant SBP reduction of -0.81 mmHg among postmenopausal women [44-46].

These results demonstrate that combined aerobic-resistance exercise training produces both clinically and statistically significant decreases in SBP, underscoring its value as a cornerstone of non-pharmacological hypertension management. Our results also show that five RCTs including 182 participants reported a significant reduction in DBP (MD= -5.87 mmHg). The homogeneity of these findings highlights the external validity of combined exercise as a scalable and sustainable non-pharmacological therapy for hypertension control. Beyond its antihypertensive effects, combined exercise may also contribute to improving body composition, metabolic profile, and overall cardiorespiratory fitness, offering broad benefits for cardiovascular prevention and rehabilitation. Evidence consistently supports the efficacy of combined aerobic and resistance exercise in lowering DBP among older adults and postmenopausal women with hypertension [42]. The meta-analysis by Li *et al*. reported a mean DBP reduction of -5.93 mmHg in elderly hypertensive patients [47]. This decline represents a clinically significant improvement, as each 5-mmHg reduction in DBP is associated with an estimated 38% decrease in stroke risk [48]. This outcome suggests that, despite age-related vascular stiffening and hormonal changes, integrated exercise modalities can still produce meaningful improvements in vascular tone and endothelial function. Although significant reductions were observed in both systolic and diastolic blood pressure, no statistically significant effect was found for mean arterial pressure. This discrepancy may be due to the limited number of studies reporting MAP and variability in exercise protocols.

Nevertheless, the observed trend toward MAP reduction supports the overall beneficial role of combined exercise. Complementary evidence from animal models supports these human findings. Da Silva *et al*. demonstrated that combining chronic physical exercise with rose oxide administration produced additive decreases in MAP in hypertensive rats, providing mechanistic insight into the synergistic modulation of vascular tone [49]. Likewise, Craig *et al*. highlighted altered MAP kinetics in hypertensive individuals during high-intensity knee-extension exercise, noting an exaggerated and delayed MAP response compared with normotensive controls [50]. These studies reveal that combined and multimodal exercise strategies consistently improve MAP control through integrated cardiovascular and endothelial adaptations, supporting their incorporation into comprehensive hypertension management protocols for both prevention and treatment. While the results are encouraging, they should be interpreted in the light of certain methodological and contextual limitations. First, many trials had small sample sizes, reducing statistical power and generalizability. Second, heterogeneity in training protocols: duration, sequence, intensity, complicates direct comparisons across studies. Third, the short duration of interventions (≤12 weeks) limits understanding of long-term adherence and sustainability of BP reductions. Finally, few studies reported on participant adherence and adverse events, which are essential for evaluating feasibility and safety in real-world settings. Despite these limitations, our review used rigorous methodological and statistical procedures, adhered to PRISMA 2020 guidelines, and included only randomized controlled trials, thereby strengthening confidence in our principal findings. The current study highlights the need to consider combined exercise into clinical and community hypertension management, particularly as a cost-effective and safe intervention for adults with hypertension.

## Conclusion

This systematic review and meta-analysis show that combined aerobic and resistance training produces meaningful reductions in systolic and diastolic blood pressure in adults with hypertension. Across randomized controlled trials, combined exercise resulted in average decreases of about -8.26 mmHg for SBP and -5.87 mmHg for DBP, supporting its value as an effective non-pharmacological adjunct to standard care. Overall, these findings reinforce current recommendations promoting structured combined exercise for hypertension management. Further RCTs with larger samples and standardized intervention protocols are needed to confirm long-term effects and refine exercise prescriptions.

### What is known about this topic

- Physical exercise is a promising non-pharmacological strategy for the prevention and management of hypertension;
- Aerobic and resistance training independently reduce systolic and diastolic blood pressure;
- Evidence regarding the combined effect of aerobic and resistance exercise on blood pressure remains heterogeneous.

### What this study adds

- Update of the current clinical practice by confirming that combined exercise produces significant and clinically relevant blood pressure reductions in hypertensive adults;
- The blood pressure reduction is consistent across diverse populations, durations, and exercise protocols, confirming its clinical relevance;
- Findings support integrating structured combined exercise into hypertension management as low-cost and effective non-pharmacological intervention.
